# Pronoun Resolution in Turkish: The Interplay of Referential Form, Word Order, and Implicit Causality

**DOI:** 10.1111/cogs.70235

**Published:** 2026-06-27

**Authors:** Duygu Sarısoy, Ebru Evcen, Joshua Hartshorne

**Affiliations:** ^1^ Department of Foreign Language Education Middle East Technical University; ^2^ Department of Linguistics University of California San Diego; ^3^ Communication Sciences and Disorders MGH Institute of Health Professions; ^4^ School of Psychology, University of Sydney

**Keywords:** Implicit causality, Anaphora resolution, Pronoun interpretation, Turkish emotion verbs

## Abstract

Pronouns are a ubiquitous part of discourse, but unusual in that their meaning is almost entirely determined by context. While early theorists hoped to explain pronouns based on a small number of simple principles, the last half‐century of research has revealed a cornucopia of influences at the syntactic, semantic, discourse, and pragmatic levels. While there are currently a few popular theories, evaluating them is complicated by the complexity of the empirical situation, which is compounded by the fact that many popular experimental methods are incommensurate and are uninterpretable under one theory or another. Moreover, with a few notable examples, research has focused on English, and so the generalizability of results is uncertain. Here, we take a step toward a clear empirical foundation for theory, with a tightly controlled study of comprehension of overt and null pronouns in Turkish. We show that pronoun resolution in Turkish is influenced by verb type, word order, and referential form, though not always in ways predicted by existing theories. Our findings highlight the need for further cross‐linguistic research and more careful experimental control in order to refine models of pronoun interpretation and better account for the interaction of syntactic and discourse factors.

## 1 Introduction

Third‐person pronouns (he/she/they/it), despite their ubiquity in discourse, pose a profound challenge for theories of language comprehension. Formally ambiguous, their interpretation depends almost entirely on contextual factors, making them a useful probe into sentence processing.

It has been widely argued that pronouns, due to being reduced referring forms, are preferentially used to refer to the most structurally prominent entity in the discourse (Ariel, 1990; Givón, 1976; Grosz, Joshi, & Weinstein, 1983; Gundel, Hedberg, & Zacharski, 1993). Conversely, full noun phrases are used to refer to less‐prominent entities, and listeners are sensitive to this fact, perhaps through implicit application of Gricean principles (“If the topic was the same, the speaker would have used a pronoun…”; Gundel et al., 1993).

The empirical status of this account is unclear, in part because researchers have differed as to whether potential referents are rendered structurally prominent due to being a sentential subject, the discourse topic, the first‐mentioned entity, or something else (Ariel, 1990; Arnold et al., 2007; Fukumura & van Gompel, 2015; Givón, 1976; Grosz et al., 1983; Gundel et al., 1993; Song & Fisher, 2007). For obvious reasons, teasing these possibilities is difficult in English (but see Fukumura & van Gompel, 2015), the language in which most studies are conducted. The more limited work in languages where these constructs can be dissociated have reached varying conclusions (Blything, Järvikivi, Toth, & Arnhold, 2021; 2022; Fukumura & van Gompel, 2015; Hert, Järvikivi, & Arnhold, 2024; Järvikivi, Van Gompel, Hyönä, & Bertram, 2005; Kaiser & Trueswell, 2008; Özge & Evcen, 2020; Schumacher, Roberts, & Järvikivi, 2017; Walker, Iida, & Cote, 1994; Yang, Gordon, Hendrick, & Wu, 1999).^1^ Moreover, this account makes a straightforward prediction that in languages where the most reduced referring expression is a null pronoun, it should be the null pronoun that refers to the most prominent entity, with overt pronouns shifting the topic (Kameyama, 1985; Prince, 1999; Turan, 1995). Again, the limited work in languages that have null pronouns has reached differing conclusions, with some studies supporting the predictions and some not (Alonso‐Ovalle, Fernández‐Solera, Frazier, & Clifton, 2002; Chamorro, 2018; Filiaci, Sorace, & Carreiras, 2014; Gelormini‐Lezama & Almor, 2011; Hartshorne, Sudo, & Uruwashi, 2013; Özge & Evcen, 2020; Papadopoulou, Peristeri, Plemenou, Marinis, & Tsimpli, 2015; Ueno & Kehler, 2016; Yang et al., 1999).

In addition to its sparsity, a likely reason that research on these questions has been inconclusive is that the effect of prominence (however, it is defined) and pronominal form interact with (at least) two other powerful, pervasive aspects of linguistic structure: discourse structure and verb semantics. This renders uninterpretable any studies of prominence or pronominal form that do not carefully control for discourse structure and verb semantics. As we review below, this is almost all of them. First, we describe the effects of discourse structure and verb semantics in more detail.

### 1.1 How discourse structure and verb semantics modulate pronoun resolution

With regard to discourse structure, Kehler (2002) catalogs over a dozen different ways two clauses may be related. Some, such as an “elaboration,” consist of a set of statements about the same thing and thus naturally involve repeated topics (*Jeremiah was a bullfrog. He was a good friend of mine*.). Others (such as “explanation” or “implicit causality” discourses) have other purposes (like providing the explanation of an event) for which topic‐preservation may be counterproductive (*The feather broke the vase. It was incredibly fragile*.). Empirically, manipulating the discourse structure has dramatic effects on pronoun interpretation (Crinean & Garnham, 2006; Hartshorne, O'Donnell, & Tenenbaum, 2015; Kehler, 2002; Kehler, Kertz, Rohde, & Elman, 2008; Stevenson, Crawley, & Kleinman, 1994). Compare, for instance, explanation discourses (1) and consequence discourses (2):

(1) Al frightened/angered/delighted Bart **because** he [= Al] drank all the wine.
(2) Al frightened/angered/delighted Bart, **so** he [= Bart] drank all the wine.

Verb semantics creates further complications. Pronoun reference is often modulated by the verb in the prior clause. Compare, for instance, (1−2) with (3−4):

(3) Al feared/hated/liked Bart because he [= Bart] drank all the wine.
(4) Al feared/hated/liked Bart so he [= Al] drank all the wine.

Note that for exposition, we chose verbs that have opposite biases in explanation and consequence discourses; however, many verbs have the same bias in both (Hartshorne et al., 2015).

Early research was unable to identify why certain verbs had the effects that they did (Garvey & Caramazza, 1974) and often resorted to post hoc categorization; many researchers, for instance, followed Rudolph and Forsterling (1997) in dividing all action verbs into two categories based entirely on their effects on pronoun resolution: *agent‐patient* verbs are those action verbs that, when used in explanation sentences, bias subsequent pronouns toward the verb's subject (*Al cheated/slandered Bart because he [= Al]…*) while *agent‐evocateur* verbs bias pronouns toward the object (*Al answered/praised Bart because he [= Bart]…*). This categorization usefully organizes verbs according to their effects on pronoun resolution; however, it remains descriptive rather than explanatory and provides little guidance for other discourse contexts (Crinean & Garnham, 2006; Pickering & Majid, 2007).

More recent work has shown a tight relationship between a verb's pronoun effect and its argument structure. Specifically, studies of thousands of verbs across a half‐dozen languages reveal that, verbs that have the same argument structure (i.e., are part of the same Levin verb class; see Appendix S1) affect pronoun biases in the same way (Hartshorne & Snedeker, 2013; Hartshorne et al., 2015; Hartshorne et al., 2013; see also Arnold, 1998; Bott & Solstad, 2014; Kehler et al., 2008).^2^ Critically, it is widely believed that verb argument structure is itself mostly or entirely driven by verb semantics (Hartshorne, Bonial, & Palmer, 2014; Levin & Rappaport Hovav, 2005). A particularly clear demonstration of this comes from Hartshorne and Snedeker (2013), who examined pronoun comprehension in explanation (“implicit causality”) discourses using a large and diverse set of verbs. Participants read sentences like *Al frightened Bart because he…* or *Al feared Bart because he…* and indicated which referent the pronoun referred to in a forced‐choice task. Rather than selecting a small number of verbs with known biases, they sampled broadly across the lexicon and estimated verb‐specific pronoun biases. They found that verbs within the same Levin verb class showed similar interpretation patterns: experiencer‐object verbs (e.g., *frighten*, *surprise*) tended to bias pronouns toward the subject, whereas experiencer‐subject verbs (e.g., *fear*, *like*) tended to bias pronouns toward the object. This provides strong evidence that fine‐grained verb semantics plays a central role in pronoun resolution, a conclusion that motivates our focus on verb type in the present study.

### 1.2 What we do and do not know about how prominence and pronominal form affect pronoun reference

The phenomena reviewed in the previous section complicate answering the empirical questions we opened with: (a) can we disentangle a preference for pronouns to refer to the previous subject versus topic (etc.), and (b) whether null pronouns in languages that have them behave like overt pronouns in languages that do not. At a minimum, there is a problem of generalization: as reviewed above, pronouns behave very differently as a function of discourse structure and verb argument structure. Thus, to make claims about subjects *in general* or null pronouns *in general*, we would ideally show that the relevant effects are seen in all combinations of discourse and argument structures, or at least in a representative sample. Moreover, subject pronouns frequently refer to something other than the previous subject—over ⅓ of the time, according to one corpus study (Arnold, 1998)—something that must be accounted for. Perhaps, for instance, there is an overarching preference for pronouns to refer to previous subjects, though this can be overwhelmed in certain contexts by effects of discourse or verb, or perhaps subjecthood only matters in certain discourse−verb combinations and is irrelevant in others (Arnold, 2023; 2025; Hartshorne et al., 2015).

Unfortunately, the design of most studies prevents them from weighing in on these questions. For instance, Fukumura and van Gompel (2015) report several experiments in English that disentangle subjecthood and order‐of‐mention, concluding that pronouns preferentially refer to the previous subject independent of whether it was first‐mentioned (*Like Bart, Al was in debt. He [= Al] …*). However, their 48 stimuli involve a variety of Levin verb classes and do not control discourse structure.^3^ Thus, the most we can say is that, of the discourse−verb combinations used, more cared about subjecthood than order‐of‐mention. Similarly, Gelormini‐Lezama and Almor (2011) compared null and overt pronouns, but controlled neither verb semantics nor discourse structure. Chamorro (2018) and Wolna, Durlik, and Wodniecka (2022) investigated pronoun form using a single discourse structure (*X when Y*) but did not control verb‐type.^4^

Perhaps the specific combination of discourse structure and verb semantics does not matter for the questions at hand. However, this cannot be assumed. The divergent findings across different studies may plausibly be explained by uncontrolled variation in precisely these factors.

The ideal study would cross pronominal form with subjecthood versus topichood (etc.) across a comprehensive (or at least representative) set of discourse structures and verb types, with enough stimuli in each condition to test for interactions. This is impractical: there are over a dozen discourse structures and hundreds of Levin verb classes. If we understood verb semantics and discourse structures better, perhaps it would be possible to find a relatively small number of conditions that are sufficient to ensure generalization, but theories of argument structure in particular remain notoriously incomplete (individual theories have been worked out for only some verbs and sentence structures, with others left for future work) and there is a great deal of disagreement between different theories (Levin & Rappaport Hovav, 2005).

If the ideal study cannot be done all at once, it will have to be done piecemeal, in a series of studies that investigate the role of subjecthood (etc.) and pronominal form in specific discourse/verb contexts. There are a small number of such studies to date. The comprehension studies among them—Hartshorne and colleagues (2013) and Özge and Evcen (2020) contradict the prediction that null pronouns refer to the most prominent entity (defined by structural properties like subjecthood, topichood, etc.) and that overt pronouns are used contrastively. Hartshorne and colleagues (2013) considered explanation (“implicit causality”) discourses in a comprehension task similar to Hartshorne and Snedeker (2013), presenting participants with sentences with an ambiguous pronoun modified by a novel adjective and asking for the referent of an ambiguous pronoun (e.g., *Sally frightened Mary because she is a dax. Who is the dax? Sally Mary*.). They found that across a number of languages (English, Mandarin, Japanese, Spanish, Dutch, Italian), experiencer‐object emotion verbs (*frighten*, *anger*) resulted in the pronoun being resolved to the subject, whereas experiencer‐subject verbs (*fear*, *hate*) had the opposite effect. Importantly, this was true of both null pronouns and overt pronouns, though they did not directly contrast null and overt pronouns in the same language. Özge and Evcen (2020) systematically probed pronoun comprehension in Turkish with a forced‐choice task similar to Hartshorne and Snedeker (2013) and Hartshorne and colleagues (2013), investigating the effects of word order, referential form, and verb valence on pronoun resolution. Participants read two‐clause sentences linked by “because,” involving physical contact action verbs (e.g., *hit*, *kick*, *kiss*), and chose which referent a nonsense adjective described. Their design crossed SOV and OSV word orders with null versus overt pronouns and also manipulated verb valence (positive vs. negative events). In Turkish, the sentence‐initial position is typically associated with the topic, whereas the preverbal position is associated with focus. They found a robust word order effect: subject interpretations increased when the subject appeared in the preverbal focus position (OSV), suggesting that focushood can boost subject's prominence in an additive fashion. At the same time, the pronominal form did not behave in a simple prominence‐tracking way. Null pronouns did not uniformly prefer the most prominent referent, and overt pronouns did not consistently signal contrast or topic shift (i.e., the interpretation of overt pronouns interacted with verb valence, with overt pronouns favoring object reference in positive events but subject reference in negative events). Instead, the effect of pronominal form varied depending on word order and verb valence. Overall, they did not find a simple mapping between pronominal form and prominence, but rather evidence that pronoun resolution reflects the interaction of grammatical role, information structure, pronominal form, and properties of the event being described.

In addition to the aforementioned studies of pronoun comprehension—the focus of our study—there is also a small literature that mixes production and comprehension measures (e.g., Frederiksen & Mayberry, 2022; Hoek, Kehler, & Rohde, 2021; Kehler & Rohde, 2019; Ng, Hwang, & Cui, 2025; Ueno & Kehler, 2016; Zhan, Levy, & Kehler, 2020). In this paradigm, participants are asked to complete sentence fragments (e.g., *Al frightened Bart because he…*). The researchers then guess who the participant intended the pronoun to refer to, which is difficult and involves some guess‐work; intercoder agreement rarely exceeds 90%. (In some cases, such as Ueno and Kehler (2016), coders must also infer the discourse structure.) Thus, the outcome measure reflects a combination of the production and comprehension biases. The precise cognitive processes this measure captures and its relationship to pronoun comprehension remain unclear (Ariel, 2013; Arnold, 2023; for a sentence‐completion method that mitigates these concerns, see Weatherford & Arnold, 2021). Note that some authors explicitly argue that production and comprehension patterns should match exactly (e.g., Hartshorne, 2014; Rosa & Arnold, 2017), whereas others argue that production is insensitive to factors that affect comprehension, such as verb semantics (Kehler & Rohde, 2019).

Among these studies, the only one to control verb semantics and discourse structure is a Mandarin study by Ng et al. (2025), which crossed null and overt pronouns with subject‐fronted and nonfronted sentences (the former increases topicality of the subject) while controlling discourse structure (result discourses, also known as “implicit consequentiality” discourses) and verb‐type (experiencer‐object verbs like *frighten* and *confuse*). Using their combined production‐comprehension measure, they found a strong overall tendency for the pronoun to refer to the previous subject, though this was stronger for topicalized subjects and for null pronouns (there was no interaction). A second experiment considered experiencer‐subject verbs^5^ like *fear* and *love*, finding near‐universal resolution of both null and overt pronouns to the previous subject when it was nontopicalized (topicalized subjects were not tested). While these findings contrast with those of Özge and Evcen (2020), it is hard to know whether that is due to differences in discourse structure, differences in verb semantics, differences in the languages, or simply because Özge and Evcen (2020) measured comprehension while Ng et al. (2025) did not.

Where does this leave us? It seems unlikely that the story is simple. The comprehension studies that do not control discourse structure or verb semantics generally show stronger subject‐biases for null pronouns than for overt pronouns, though the latter are often subject‐biased as well. The more controlled studies suggest that there are circumstances in which null pronouns are reliably resolved to the previous object (not subject) and where overt pronouns are more subject‐biased than null pronouns. The less controlled studies vary in what structural properties (subjecthood, topichood, etc.) are relevant, and the one controlled study points to focus over topichood, though broad generalizations are not yet possible given the small number of such studies. The best course of action, it would seem, is to run more controlled studies until this picture is sufficiently clear to propose generalizations that can then be tested. In the present paper, we address two additional contexts in Turkish.

### 1.3 Pronoun resolution in Turkish

Turkish provides a nice opportunity to study the questions of structural prominence and pronominal form. As a flexible word‐order language, it provides opportunities for distinguishing grammatical effects from discourse‐semantic factors in pronoun resolution. Turkish is an SOV (head‐final) language with flexible word order. Word order in Turkish systematically encodes information structural elements, where the sentence‐initial entity is typically the topic, while the preverbal entity receives the focus position (Erguvanlı‐Taylan, 1986; Erkü, 1983; İşsever, 2003).

These features provide a particularly useful ground for dissociating the effects of subjecthood, topic/focus‐hood, and first‐mention bias. Along with word order variation, Turkish also allows the drop of both subject and object arguments and their corresponding pronouns.

Theorists have been divided as to how these factors ought to affect pronoun interpretation. According to Kerslake (1987), Turkish null subject pronouns refer to the previous subject, though overt pronouns can as well if contrastive stress is used. Erguvanlı‐Taylan (1986) suggests that this depends on clause type: the object tends to appear as an overt pronoun in a subordinate clause, but it could appear both as an overt or a null pronoun in a conjoined clause. In contrast, Enç (1989) argues that null pronouns refer to the previous topic (the sentence‐initial noun phrase), whereas overt pronouns shift the topic and mark the contrast in Turkish and thus should refer to the nontopic.

None of the aforementioned work has considered the role of discourse structure. Concerning the role of verb‐semantics, Turan (1997) made an initial theoretical observation that although null pronouns usually refer to a previous subject entity, this tendency can shift in emotion verbs, in which case the experiencer becomes more salient and may attract pronominal reference. Accordingly, this tendency interacts with a thematic hierarchy ranking the agents higher than experiencers and ranking the experiencers higher than arguments with the theme (or stimulus) role. Thus, a subject bearing an agent role will be more discourse salient compared to object themes; an object in the experiencer role in emotion verbs will be more salient than a subject in the theme (stimulus) role.

As noted above, Özge and Evcen (2020) experimentally investigated pronoun resolution in Turkish and showed that information structure plays a clear role. In particular, they found more subject interpretations in OSV orders than in SOV orders, suggesting that a referent becomes especially salient when it is both the subject and in the preverbal position. Importantly, patterns of pronominal form resisted a simple prominence pattern: null pronouns did not always refer to the most prominent referent, and overt pronouns did not always mark topic shift. This finding underscores the role of information structure and challenges earlier theoretical claims that null pronouns simply track the topic entity (e.g., Enç, 1989; Turan, 1995). The generality of this pattern remains to be tested, as Özge and Evcen (2020) examined only physical contact events in causal discourses. Thus, whether similar effects would be observed in implicit causality contexts remains an open empirical question.

## 2 Present study

Based on the review above, we can distinguish two questions:

Q1. Do null pronouns refer to structurally prominent entities, with overt pronouns contrastively referring to nonprominent entities?
Q2. Is the primary determinant of structural prominence in Turkish subjecthood, topichood, or focus?

The results of Özge and Evcen (2020) suggest that “the general expectation to link the null pronoun to the subject (topic) referent while linking the overt pronoun to the object (nontopic) referent is not an across‐the‐board situation but it is modulated by multiple factors such as word order, the type of anaphoric expression and the verb valence among others” (p. 181). However, as also reviewed above, we cannot rule out the possibility that this conclusion is specific to physical contact verbs used in explanation (“implicit causality”) discourses. Prior researchers have variously argued that structure prominence is due to subjecthood or topichood, and some have suggested that overt pronouns are primarily used to refer to nonprominent entities (Ariel, 1990; Arnold, 1998; Givón, 1983; Gordon, Grosz, & Gilliom, 1993; Gundel et al., 1993; Kaiser & Trueswell, 2008). Perhaps this is because there are contexts in which this is true.

We thus conducted a study extending the work of Özge and Evcen (2020). We extend in two ways. First, we use different verbs from a different semantic domain, allowing us to see whether their results were specific to physical contact verbs. Second, we contrast two classes of verbs that are expected to have strongly contrasting effects of pronoun interpretation. Ideally, we would use Levin verb classes (Appendix S1). Unfortunately, these are only fully described for English and Czech (Pala & Horák, 2008). Thus, following Hartshorne et al. (2013), we focus on emotion verbs, which are relatively easy to identify and have shown reasonably uniform behavior in languages spanning four language families (Hartshorne et al., 2013). Turkish psychological state verbs have also been systematically classified according to their argument structural and aspectual features (Ibe‐Akcan, 2004), which constitutes an independent basis of our selection of items in the present study. We, therefore, take the verbs in our study to belong to the same Levin verb class, although a comprehensive Levin‐style analysis of Turkish verbs is not available. Specifically, there are two classes of transitive emotion verbs: experiencer‐subject verbs (*like*, *love*, *hate*), which are object‐biased in explanation (“implicit causality”) discourses in every language tested to date (Turkish has not been experimentally tested), and experiencer‐object verbs (*frighten*, *delight*, *anger*), which are subject‐biased.

Otherwise, we follow Özge and Evcen (2020) closely. We again cross pronominal form with word order. As explained above, the word order manipulation allows us to investigate topichood and focus through word order, contrasting SOV (subject‐topic, object‐focus) and OSV (subject‐focus, object‐topic), both of which are reasonably natural. We use the same forced‐choice comprehension method modeled after Hartshorne and Snedeker (2013), described below. In summary, we use a 2 (OSV vs. SOV) × 2 (null pronoun vs. overt pronoun) × 2 (experiencer‐subject verbs vs. experiencer‐object verbs) design.

### 2.1 Predictions

This is the first study of pronoun comprehension in any language to test the interaction of verb semantics, word order, and pronominal form using emotion verbs in implicit causality contexts. Based on the empirical work reviewed above, we expect all three to play a role in pronoun resolution. The central question is whether they interact.

One possibility is that these factors do not interact, but instead contribute independently to interpretation. In that case, their effects should be purely additive. We might expect across the board, that (i) null pronouns refer to the subject/topic and overt pronouns refer to the object/nontopic (following Enç, 1989; Erguvanlı‐Taylan, 1986; Turan, 1995); (ii) pronouns refer to the STIMULUS rather than the EXPERIENCER (the subject of *frighten* but the object of *fear*; Fedele & Kaiser, 2015; Hartshorne et al., 2013; Ueno & Kehler, 2016; but see Román, 2020), or (iii) to the EXPERIENCER rather than the STIMULUS (Turan, 1997).

Some empirical support for additive effects comes from Özge and Evcen (2020), who reported that pronominal reference was influenced by the information structure, such that they found greater subject preference in the OSV order (i.e., when the subject was in the focus position) compared to the SOV order, regardless of the pronominal type. Also consistent with this view, Ng et al. (2025)—using a production‐based measure—found that both null and overt pronouns referred to the previous subject, but the effect was stronger for null pronouns. Similarly, across the board, topicalization of the subject resulted in more pronoun reference to the subject.^6^ They also found more references to the EXPERIENCER than the STIMULUS, but that is the expected pattern because they used result (“implicit consequentiality”) discourses. Interestingly, though, in every condition, pronouns were resolved to the subject more than the object.

A second possibility is that these factors interact rather than contributing independently. Perhaps, for instance, the focus is only preferred when it also bears the STIMULUS role (which attracts pronoun resolution in explanation discourses). Perhaps, this also interacts with the referential expression (e.g., null pronouns preferring the focus, whereas overt pronouns disprefer it) or with the verb semantics (e.g., STIMULUS of experiencer‐subject verbs prefer null pronouns). Theories to date are largely silent on these questions, because they do not address all three factors. Turan (1997), for instance, theoretically predicts that the EXPERIENCER in Turkish will be more prominent than STIMULUS. Because Turan does not add any qualifiers, by implication, the effect does not interact with other factors as it does in English (specifically, discourse structure; see Crinean & Garnham, 2006; Hartshorne et al., 2015). However, Turan (1997) does not discuss discourse structure or the English data and so does not explicitly endorse an additive account.

A third possibility is that some of these factors play little or no role in Turkish pronoun resolution. Many theorists have argued that null pronouns reliably refer to the previous subject (or topic), whereas overt pronouns reliably do not (Ariel, 1990; Enç, 1989; Givón, 1976; Gundel et al., 1993; Kerslake, 1987).

Note that these broad possibilities give rise to a variety of subhypotheses and subpredictions. For instance, we may find that in this context (unlike in Özge & Evcen, 2020), topic attracts pronoun resolution more than does focus. We might find that, as suggested by Turan (1997), the EXPERIENCER attracts pronoun resolution, not the STIMULUS. And so on. This uncertainty highlights the degree to which studies like the present one are needed, as is theoretical work that explicitly considers all the factors known to affect pronoun resolution.

### 2.2 Methods

All materials, data, and analysis code for this and all the following experiments are available at https://osf.io/jn9fh .

#### 2.2.1 Participants

One hundred and thirty‐six native speakers of Turkish, who were all undergraduate students in Turkey, completed the study. No participants who completed the study were excluded, because none met our exclusion criterion of choosing the same referent across all conditions, which would be a sign of not paying attention. Twenty‐nine participants were randomly assigned to the first set, 37 participants to the second set, 32 participants to the third set, and 38 participants to the final set. The imbalances in sample sizes across the four lists were due to the participants starting the experiment but not completing it.

#### 2.2.2 Materials and procedures

We designed a 2×2×2 study where we manipulated the word order (SOV, OSV), referential form (null vs. overt pronoun) between‐participants, and verb type (experiencer‐subject, experiencer‐object verbs) within‐participants. We selected the emotion verbs that exhibited the strongest implicit causality effects in Hartshorne and Snedeker (2013) and translated them into Turkish. In selecting Turkish emotion verbs, we also consulted descriptions of Turkish psych verb classes (e.g., Ibe‐Akcan, 2004) and restricted our materials to verbs that take nominative subjects and accusative objects, with animate subject–animate object combinations. We excluded verbs with overt causative morphology (e.g., korkut‐ “frighten”) as well as experiencer‐subject verbs that require nonaccusative case marking (e.g., kork‐ “fear,” which takes an ablative‐marked stimulus), so that verb‐type contrasts would not be confounded with differences in overt morphology and case marking. From these, we selected eight experiencer‐subject‐type verbs (onayla‐/favor, arzula‐/desire, beğen‐/like, sev‐/love, affet‐/pardon, kıskan‐/envy, aşağıla‐/disdain, yargıla‐/judge) and eight experiencer‐object‐type verbs (büyüle‐/fascinate, cezbet‐/attract, etkile‐/dazzle, sık‐/bore, kır‐/offend, üz‐/upset, yarala‐/hurt, örsele‐/mistreat). A sample item for each condition was presented in (3) and (4):

**Table:** 

(3)	a	SOV, Zero/Overt pronoun, Experiencer‐Subject/Experiencer‐Object verb					
		Bahar	Ceren'i	arzulu‐yor/büyülü‐yor	çünkü	(o)	dakmuk.
		Bahar‐nom	Ceren‐acc	desire/dazzle‐prog.3sg	because	(she)	dakmuk
		“Bahar desires/dazzles Ceren because she is dakmuk.”					
(4)	a	OSV, Zero/Overt pronoun,Experiencer‐Subject/Experiencer‐Object verb					
		Bahar’ı	Ceren	arzulu‐yor/büyülü‐yor	çünkü	(o)	dakmuk.
		Bahar‐ acc	Ceren‐_nom_	desire/dazzle‐prog‐3sg	because	(she)	dakmuk
		“Ceren desires/dazzles Bahar because she is dakmuk.”					

We presented participants with a sentence composed of a main clause and a subordinate clause conjoined with ‘*because*.’ The main clause was composed of two referents with [+human] and [+female] features for the subject and the object entity (e.g., Sally frightens Mary). The subordinate clause was constructed with an ambiguous pronoun and a nonword adjectival predicate ‘*dax*’(e.g., …because she is dax). We asked participants to decide who the referent for the nonword is, following previous empirical work (e.g., Hartshorne & Snedeker, 2013; Özge & Evcen, 2020). This made participants resolve the ambiguous pronoun toward the subject or the object without explicitly asking them who the pronoun refers to.

We created four counterbalanced and pseudorandomized lists with 16 critical (eight experiencer‐subject‐type, eight experiencer‐object‐type) and 24 filler items. The filler items included other verb types such as physical touch action verbs, reciprocal verbs, and benefactives. We also counterbalanced the order of the answer options (*Bahar*, *Ceren*) for the question (*Who is dakmuk?*) so that each referent appeared equally often.

We collected offline responses from participants through a web‐based survey tool. Each trial was presented one at a time to prevent participants from making comparisons between their judgments and changing their answers. Participants used their phones in a classroom setting, and they all completed the task individually. The experiment took approximately 15 min to complete.

## 3 Results

**Fig. 1:**
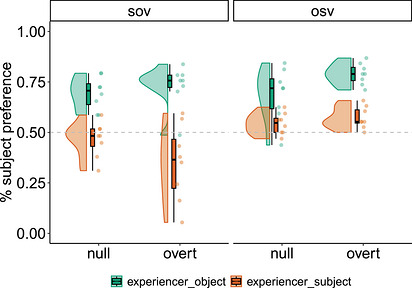
Proportion of choice of subject for each Word Order (SOV, OSV), Referential Form (null, overt), and Verb Type (experiencer‐subject‐type, experiencer‐object‐type). Each dot represents an item. Horizontal dashed line indicates selection at chance.

Fig. 1 shows the proportion of subject choices for each combination of Word Order, Referential Form, and Verb Type. Individual verb results are presented in Appendix S2.

We begin by testing the most robust semantic prediction, namely, that verb type should influence pronoun resolution. To confirm an effect of verb bias, we conducted a pairwise *t*‐test comparing subject preference scores between the two verb groups. The results indicated a significant difference between experiencer‐object verbs and experiencer‐subject verbs (*t*(260) = −8.23, *p* < .001). Follow‐up one‐sample *t*‐tests revealed that experiencer‐object verbs exhibited a strong subject bias, with scores significantly greater than chance (*M* = 72.88, *SD* = 0.22, *t*(135) = 12.01, *p* < .001), while experiencer‐subject verbs did not show a significant bias toward either subject or object (*M* = 48.16, *SD* = 0.27, *t*(135) = −0.79, *p* = .40). Thus, in this comprehension task, the two verb classes pattern differently in Turkish in a way that is consistent with the cross‐linguistic distinction between experiencer‐object and experiencer‐subject verbs. Although these verb classes were identified based on prior cross‐linguistic work, the present results themselves provide evidence that Turkish speakers distinguish these classes in pronoun interpretation, with experiencer‐object verbs showing a strong subject (i.e., STIMULUS) bias relative to experiencer‐subject verbs.

We next asked whether the effects of verb semantics, word order, and referential form combine additively or interact. To do so, we submit the data to a partially Bayesian generalized mixed effects logistic regression with Wishart priors on the covariance matrix for random effects using the *blme* package with bobyqa optimization in order to improve convergence and avoid issues with singularity (Chung, Rabe‐Hesketh, Dorie, Gelman, & Liu, 2013). Models were constructed to predict subject preference from contrast‐coded fixed effects of Word Order (SOV, OSV), Referential Form (null, overt), and Verb Type (experiencer‐subject‐type, experiencer‐object‐type), including their interactions. We began with a model that included maximal effects structure and refined the model, removing noncontributory variance components until no further improvements were observed via likelihood ratio tests (Bates et al., 2015). Our final model included as main effects all three fixed effects and the interaction of Verb Type and Referential Form and Word Order and Referential Form (see Appendix S3 for model selection details). Random effects include random intercepts of subject and item as well as, and random slopes of Verb Type by subject and Word Order by item. We report the output of the best‐fitting model regression coefficient, standard error, Wald's *z*‐value, and *p*‐value.^7^

The model revealed a significant main effect of Verb Type (β = 1.21, *SE* = 0.18, *z* = 6.62, *p* < .001) such that subject responses were produced significantly more often for experiencer‐object‐type verbs (*M* = 72.88; 95% CI [69.11, 76.65]) than experiencer‐subject‐type verbs (*M* = 48.16; 95% CI [43.57, 52.74]) and an effect of Word Order (β = 0.52, *SE* = 0.24, *z* = 2.49, *p* = .02), reflecting a larger subject preference in OSV order (*M* = 65.08; 95% CI [60.51, 69.65]) than in SOV order (*M* = 55.68; 95% CI [50.61, 60.75]). These main effects are broadly consistent with an additive view in which both verb semantics and information structure independently bias pronoun resolution. We also observed a significant interaction between Verb Type and Referential Form (β = 0.61, *SE* = 0.28, *z* = 2.16, *p* = .03). The difference between the two levels of Referential Form is not statistically significant at either level of verb type, although the direction and magnitude of the effect are different: For the experiencer‐subject‐type verbs, the subject preference was nonsignificantly stronger for null pronouns compared to overt pronouns (46.2 [39.7, 52.6] vs. 50.6 [44.1, 57.2]; *p* = .3), whereas the reverse was true for experiencer‐object‐type verbs (76.1 [71.4, 80.6] vs. 69.1 [62.8, 76.3]; *p* = .07). Given that the pairwise comparisons were not significant, the interaction was barely significant, and the direction of the effect admits no obvious interpretation, Occam's razor suggests that the interaction is Type I error. We do not discuss it further. There were no other significant effects.

## 4 General discussion

Our aim in this study was to investigate how the factors like information structure encoded in word order variation (SOV vs. OSV), the type of anaphoric expression (null vs. overt pronouns), and verb semantics (implicit causality) influence the interpretation of pronouns in Turkish. In our study, participants read an utterance constructed with an emotion verb, followed by another clause connected with a causal connective “because.” The second clause ended with a nonsense adjectival predicate “dakmuk.” Given a binary choice between the two referents (Subject, Object) of each utterance, the participants determined the antecedent of this adjectival predicate.

Across all conditions, participants were more likely to resolve the pronoun to the subject for experiencer‐object‐type verbs than experiencer‐subject‐type verbs. There was a slightly stronger tendency to resolve pronouns to the previous subject in OSV order relative to SOV, which numerically was more pronounced for experiencer‐subject‐type verbs. Critically, however, we did not find reliable interactions. This is consistent with the additive view in which the verb type and word order effects combine cumulatively. These results contrast clearly with the predictions of Turan (1997), who suggests the opposite effect for verb‐type; an interaction of verb‐type with reference type, which we did not find; and no effect of word order, which we did find. In retrospect, it seems likely that Turan's (1997) predictions about verb‐type were influenced by the nature of her data. Turan's (1997) study used examples from naturally occurring written data, where the animacy across the verb type was not controlled. In her examples, the experiencer was animate, while the stimulus was inanimate, which may have biased participants to expect pronouns to refer to the (animate) EXPERIENCERS. In contrast, our study controlled animacy. We do not currently have a hypothesis as to why Turan's (1997) investigation suggested that reference type interacts with verb‐type. We return to the word‐order results below.

Our data are largely consistent with what would be predicted from the implicit causality literature: both overt and null pronouns are resolved to the previous STIMULUS. (This literature made no clear prediction about word order, which we return to below.) The primary caveat is that there was an overall subject bias (in most conditions, the experiencer‐subject‐type verbs are roughly equi‐biased rather than being strongly object‐biased), which is not quite what implicit causality research would tend to suggest. However, across implicit causality studies, there is quite a bit of variability in whether studies show overall subject preferences, overall object preferences, or no preference (for instance, compare Hartshorne & Snedeker, 2013, with Hartshorne et al., 2015). The reasons remain unclear and require further investigation.

The existence of a word‐order effect was not predicted by the implicit causality literature, which makes no clear predictions about information structure, and it contrasts with Turan's (1997) expectation that word order should not affect pronoun interpretation. In that sense, the effect we found—an increase in subject preference in OSV—was unexpected from a theoretical standpoint, though not without empirical precedent: a similar pattern was reported for physical contact action verbs in Özge and Evcen (2020). In both studies, subject interpretations increased when the subject appeared in the preverbal focus position, suggesting that focushood can enhance the prominence of a referent beyond grammatical role alone. Thus, while the direction of the effect is consistent with prior experimental findings in Turkish, it remains theoretically surprising because most prominence‐based accounts predict that pronouns should favor topics or subjects, not focused elements (e.g., Ariel, 2013; Grosz et al., 1983; Hoffman, 1998). One possible explanation is an overadditive interaction of subjecthood and focushood. The effect is relatively small, so for the moment, we do not wish to overinterpret it. However, it underscores the complexity of pronoun resolution and the extent to which classic theories lack mechanisms to account for the interaction of grammatical role and information structure. Future studies should control grammatical role, information structure, and discourse context rather than treating subjecthood (or any other marker) as a sufficient marker for prominence. Also, cross‐linguistic studies should investigate whether similar subject‐focus overadditive effects arise in other discourse configurational languages where preverbal focus position is available. Finally, theoretical models of prominence may need to incorporate explicit hypotheses regarding the mechanisms for the interaction between grammatical role and focus status rather than ranking these effects independently.

One important limitation is that our study did not manipulate or control prosody, which is known to influence pronoun interpretation (e.g., Cowles, Walenski, & Kluender, 2007). In natural speech, prosodic prominence may interact with information structure and referential form in ways that could amplify or attenuate some of the effects observed here. More generally, the history of research on pronoun resolution shows that new and sometimes unexpected factors continue to emerge. It is, therefore, possible that additional, as yet undescribed variables also contribute to the patterns we report. As concrete next steps, future work should directly manipulate prosody, test additional coherence relations beyond because‐clauses, and broaden verb sampling beyond emotion verbs to assess how general the word‐order effect is. Continued experimental and exploratory work across different constructions, verb types, and discourse contexts will be essential for building a more complete theory of pronoun interpretation.

## 5 Conclusion

Anaphora has been a core topic of study for the language sciences for decades. It has become increasingly clear that simple theories are inadequate to capture the complexity of the phenomenon. Unfortunately, the discovery of this complexity also makes it difficult to interpret many earlier studies, which do not control for factors that are now known to be critical. Here, we take advantage of the affordances of Turkish to investigate the effects of reference type (null vs. overt pronouns) and word order, focusing on two verb‐types and one discourse structure. As one might expect at this point, we find some unexpected results. Clarifying the factors underlying pronoun resolution will require many such studies.

## Conflicts of interest

Duygu Sarısoy declares that she has no conflicts of interest. Ebru Evcen declares that she has no conflicts of interest. Joshua Hartshorne declares that he has no conflicts of interest.

## Ethics statement

All procedures performed in studies involving human participants were in accordance with the ethical standards of the institutional and/or national research committee and with the 1964 Helsinki declaration and its later amendments or comparable ethical standards. This study was approved by the Ethics Committee of the Middle East Technical University (protocol no 0025‐ODTUIEAK‐2025).

## Supporting information



Supporting Information

## Notes

1 For instance, as we explain below, Turkish uses word‐order, rather than syntactic function, to indicate topic.

2 Note that this is a more specific claim than what is made by some earlier.

3 Their stimuli did not include any explicit discourse connectives (because, so, etc.), rendering the discourse structure underspecified. This may result in different interpretations of the sentence and of the pronoun specifically (Kehler, 2002; Kehler, Kertz, Rohde, & Elman, 2008). To date, the only available method for determining the discourse structure is the use of trained coders.

4 The author reports that the verbs “did not bias any particular interpretation,” but it is not clear how that was determined or even how it could be determined, given that verb bias depends on discourse structure.

5 Note this spans a half‐dozen Levin verb classes. In most studies that use this terminology, the majority of experiencer‐stimulus verbs come from two Levin classes, both of which have strong object biases in explanation (“implicit causality”) discourses: transitive emotion verbs (*fear*, *like*) and intransitive emotion verbs (*marvel at, worry about*) (Hartshorne & Snedeker, 2013; Hartshorne, O'Donnell, & Tenenbaum, 2015). However, researchers sometimes include various types of perception verbs (*detect, hear*) and cognition verbs (*know, misinterpret, misunderstand*), among others. Whether such verbs are reliably object‐biased as well remains unclear, especially outside of English. Ng and colleagues do not specify what verbs they used.

6 Note that the word order manipulation used by Özge and Evcen (2020), which we adopt here, does not test the effect of topicalization. This differs from Ng, Hwang, and Cui (2025), who studied explicit topicalization by fronting the subject to the sentence initial position. In Özge and Evcen's (2020) design, the comparison was between canonical SOV orders, where the subject occupied the topic position, and OSV orders, where the object occupied the topic position. Within this contrast, locating the subject in the preverbal focus position increased its salience, while locating the object in the topic position in OSV orders or in the focus position in SOV order did not yield comparable effects.

7 A model with all three two‐way interactions fit slightly better (*p* = .04). However, neither of the other two two‐way interactions were individually significant in either a log‐likelihood test or using Wald's *z*. Thus, for simplicity of description, we emit them. For details on this model—which supports the same conclusions as the one described here—see Appendix S3 for model selection criteria.
