# Supplementary material for: Pronoun Resolution in Turkish: The Interplay of Referential Form, Word Order, and Implicit Causality
Source: Cogn Sci. 2026 Jun 27;50(6):e70235. doi: 10.1111/cogs.70235 (PMC13309914; doi:10.1111/cogs.70235)
Supplement: Supplementary file 1 — Supporting Information [file COGS-50-e70235-s001.pdf]

Appendix 1: Levin verb classes.

Different verbs can appear in different sentence structures (compare: *Al feared/hated/liked that Bart drank the wine* with *Al *frightened/*angered/*pleased that Bart drank the wine*). Linguists have identified hundreds of sentence structures in English. Most verbs are grammatical in only a handful. When verbs are classified according to which structures they can be used in, the result is not a uniform distribution; rather, there are classes of verbs that behave in the same way (Levin, 1993). Strikingly, verbs in these classes seem to be semantically related (Levin, 1993; Hartshorne et al., 2014). For instance, one class is comprised of transitive experiencer-subject emotion verbs (*fear, love*, *hate*) whereas another is comprised of transitive experiencer-object emotion verbs (*frighten*, *delight*, *anger*). Several hundred Levin verb classes have been identified for English and have been compiled into VerbNet, a free online database (Kipper et al., 2006). Unfortunately, with the exception of Czech, similar resources do not exist for other languages. However, researchers appear to assume that similar classes exist in all languages. For initial forays in other languages, see: Aparicio et al., 2008; Estarrona et al., 2016; Liu, 2020; Pradet et al., 2014).

Appendix 2: Verb by Verb Subject Bias

**Class**	**Verb**	**Subject bias**
experiencer-subject	beğen- 'like'	37%
experiencer-subject	sev- 'love'	40%
experiencer-subject	onayla- 'favor'	45%
experiencer-subject	arzula- 'desire'	47%
experiencer-subject	affet- 'pardon'	51%

experiencer-subject	yargıla- ‘judge’	51%
experiencer-subject	kıskan- ‘envy’	54%
experiencer-subject	aşağıla- ‘disdain’	59%
experiencer-object	örsele- ‘mistreat’	61%
experiencer-object	cezbet- ‘attract’	71%
experiencer-object	sık- ‘bore’	72%
experiencer-object	büyüle- ‘fascinate’	73%
experiencer-object	kır- ‘offend’	74%
experiencer-object	yarala- ‘hurt’	76%
experiencer-object	etkile- ‘dazzle’	78%
experiencer-object	üz- ‘upset’	79%

Appendix 3: Model Selection

All models can be viewed at the following: https://osf.io/jn9fh

The dependent variable in our analysis is the binary proportion of subject/object responses. The fixed effects considered include *Word Order, Verb Type* and *Referential Form*. Our model selection process began with a random effects-only model that included full 3-way interaction.

Starting with the random effects-only model, we incrementally added random slopes to assess if they significantly improved the model fit. The inclusion of all slopes, such as Verb Type by participant and *Referential Form* and *Word Order* by item significantly improved the model fit ($\chi 2(11) = 27.17$, $p < .01$).

To check whether all random slopes are necessary, we first added *Verb Type* by participant to the base random effects-only model, which improved the fit ($\chi2(2) = 7.3$, $p < .05$). Next, we removed *Referential Form* by item from the model with all slopes and found that it did not significantly affect the fit ($\chi2(2) = 1.54$, $p = .46$). However, removing *Word Orde*r by item from the model did significantly worsen the fit, so we decided to retain this slope ($\chi2(2) = 11.44$, $p < .01$).

We then added *Verb Type* by participant to the model that already included Word Order by item, which resulted in a better fit ($\chi2(2) = 8.04$, $p < .05$). Similarly, adding Word Order by item to a model with *Verb Type* by participant also improved the fit ($\chi2(2) = 12.19$, $p < .01$). Thus, the best random effects structure was determined to be (1+verb_type|participant) + (1+word_order|item) ($\chi2(4) = 19.49$, $p < .001$).

We then compared a two-way interaction model with a three-way interaction model, finding that the three-way interaction did not provide a better fit ($p < .6$). To determine the necessity of each two-way interaction, we found that removing all two-way interactions worsened the model fit ($\chi2(3) = 12.78$, $p < .001$), so we decided to keep two-way interactions but trim the non-contributory effects. Specifically, dropping the *Referential Form*Verb Type* interaction worsened the fit ($\chi2(1) = 6.01$, $p < .05$), and *Verb Type*Word Order* ($\chi2(1) = 3.95$, $p < .05$)whereas removing the *Word Order*Referential Form* interaction ($p = .09$) did not significantly change the model.

Consequently, the model with all the main effects and the *Referential Form*Verb Type* and *Word Order*Referential Form* interaction was better than a model with only main effects ($\chi2(1) = 6.09$, $p < .05$). However, this model was still less optimal than the model with all two-way

interactions ($\chi2(2) = 6.68$, $p < .05$). Given that the significant effects were consistent in both the model with all two-way interactions and the model with only the *Referential Form*Verb Type* and *Word Order*Referential Form* interaction, we decided to report the simpler model as it supports the same conclusions in Table 1 (but see Table 2 for the output of the model with all two-way interactions). Thus, our final model structure is ~ word_order*referential_form + verb_type*referential_form + (1+verb_type|participant) + (1+word_order|item).

Table 1. Model output

~ word_order*referential_form + verb_type*referential_form + (1+verb_type|participant) + (1+word_order|item)

	**Estimate**	**SE**	**z value**	**p value**
(Intercept)	0.55	0.12	4.68	<.001
verb_type	1.21	0.18	6.62	<.001
referential_form	0.11	0.2	0.57	.57
word_order	0.52	0.23	2.24	<.05
verb_typeC:referential_form	0.61	0.28	2.16	<.05
referential_formC:word_order	0.66	0.4	1.63	.1

Table 2. Model output

~ word_order*referential_form + verb_type*referential_form + word_order*verb_type + (1+verb_type|participant) + (1+word_order|item)

	**Estimate**	**SE**	**z value**	**p value**
(Intercept)	0.56	0.12	4.87	<.001

verb_type	1.31	0.18	7.29	<.001
referential_form	0.09	0.19	0.49	.6
word_order	0.48	0.23	2.1	<.05
word_order:verb_type	-0.6	0.34	-1.89	.06
verb_type:referential_form	0.6	0.27	2.16	<.05
word_order:referential_form	0.7	0.40	1.63	.1

Appendix 4: Exploratory Analyses - The Effect of Emotional Valence

As an exploratory analysis, we investigated whether the emotional valence of verbs influenced the interpretation of ambiguous pronouns. Emotional valence was categorized as either positive (e.g., *sev-/love*) or negative (e.g., *üz-/upset*). In the experiencer-subject-type condition, there were eight verbs: five with positive valence (*onayla-/favor, arzula-/desire, beğen-/like, sev-/love, affet-/pardon*) and three with negative valence (*kıskan-/envy, aşağıla-/disdain, yargıla-/judge*). Similarly, for the experiencer-object-type condition, there were eight verbs: three with positive valence (*büyüle-/fascinate, cezbet-/attract, etkile-/dazzle*) and five with negative valence (*ık-/bore, kır-/offend, üz-/upset, yarala-/hurt, örsele-/mistreat*). Due to the limited number of items within each group, the analyses were constrained, and the findings should be interpreted as preliminary, warranting more rigorous investigation.

We added *Verb Valance* as a contrast-coded fixed effect factor alongside other fixed factors, such as *Referential Form*, *Verb Type*, and *Word Order*, and followed the same model selection criteria described in Appendix 3. The best-fitting model included *Verb Type* by participant and *Word Order* by item as random slopes. Fixed effects in the model included interactions between

Verb Valance and *Verb Type*, *Verb Type* and *Referential Form* as well as *Word Order* and *Verb Type*. Consistent with findings from the main analyses, the model revealed a main effect of *Verb Type* (β = 1.28, SE = 0.16, z = 7.57, $p < .001$), with subject responses occurring more frequently for experiencer-object-type verbs than experiencer-subject-type verbs, and *Word Order* (β = 0.46, SE = 0.22, z = 2.03, $p < .05$), indicating a stronger subject preference in OSV order compared to SOV order as well as an interaction between *Verb Type* and *Referential Form* (β = 0.55, SE = 0.27, z = 2.02, $p < .03$), *w*ith no significant difference between pronoun conditions for experiencer-subject-type verbs, but a marginally higher subject preference for overt pronouns in experiencer-object-type verbs . Crucially, there was a significant interaction between *Verb Valance* and *Verb Type* (β = -0.68, SE = 0.26, z = -2.57, $p < .01$), such that, in the experiencer-subject-type verbs, negative valence verbs showed a stronger subject preference compared to positive valence verbs (β = -0.5, SE = 0.19, z = -2.64, $p < .01$). However, for experiencer-object-type verbs, no significant differences were observed based on valence ($p = .31$) (see Figure 2). These findings highlight the potential influence of emotional valence in certain verb contexts, though further research with a larger set of stimuli is needed to confirm these observations.

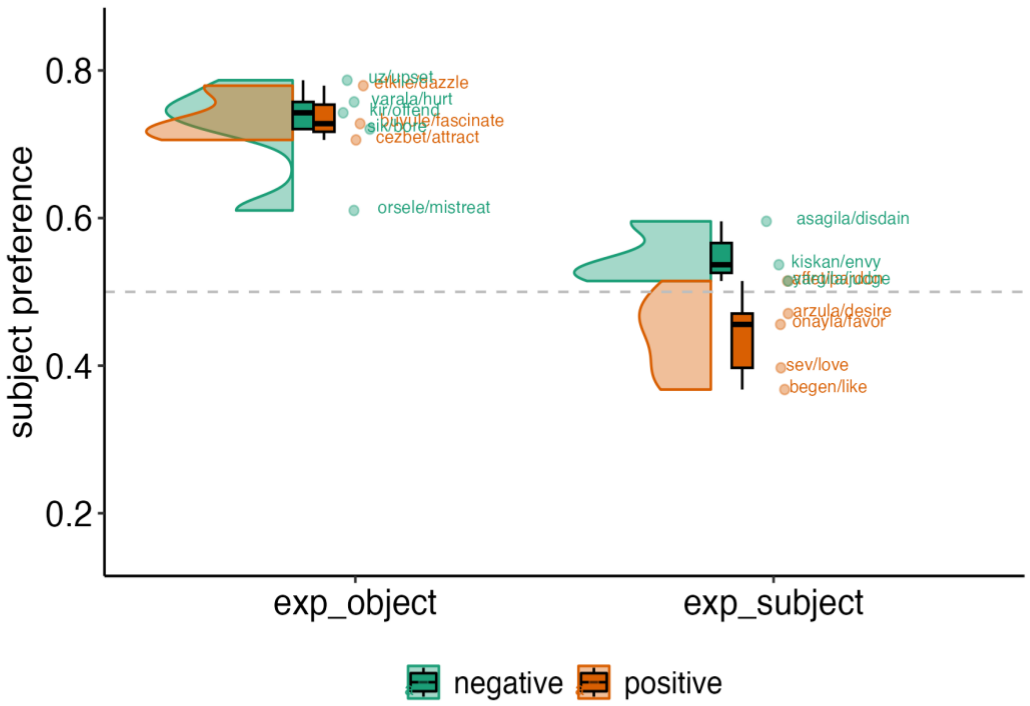

Figure 2. Proportion of choice of subject for each Verb Type (experiencer-subject-type, experiencer-object-type) by Emotional Valence (positive, negative). Each dot represents a verb. Horizontal dashed line indicates selection at chance.
